# Selenocysteine derivative overcomes TRAIL resistance in melanoma cells: evidence for ROS-dependent synergism and signaling crosstalk

**DOI:** 10.18632/oncotarget.2008

**Published:** 2014-05-26

**Authors:** Wenqiang Cao, Xiaoling Li, Shanyuan Zheng, Wenjie Zheng, Yum-shing Wong, Tianfeng Chen

**Affiliations:** ^1^ Department of Chemistry, Jinan University, Guangzhou, China; ^2^ School of Life Sciences and State Key Laboratory of Agrobiotechnology, The Chinese University of Hong Kong, Hong Kong S.A.R., China

**Keywords:** Chemosensitizer, TRAIL, Selenium, apoptosis, ROS, p53

## Abstract

Tumor necrosis factor–related apoptosis-inducing ligand (TRAIL), as one of the most promising targeted drug for new cancer therapeutics, is limited in clinical application by the evolution of resistance in many cancer cell lines, especially in malignant melanoma. Thus, it is urgently needed to identify chemosensitizers to enhance the apoptotic inducing efficacy of TRAIL and overcome resistance of malignant melanoma cells. Herein, we reported that 3,3′-diselenodipropionic acid (DSeA), a Selenocysteine derivative, could synergistically enhance the growth inhibitory effect of TRAIL on A375 melanoma cells though induction of ROS-dependent apoptosis with involvement of PTEN-mediated Akt inactivation and DNA damage-mediated p53 phosphorylation, which subsequently activated mitochondrial and death receptor apoptotic pathways. Moreover, silencing of p53 down-regulated the expression levels of p53-inducible genes, and effectively blocked the cell apoptosis. Suppression of PI3K significantly increased the apoptotic cell death. In contrast, antioxidants effectively reversed the cell apoptosis through regulation of Akt and p53 signaling pathways. Taken together, the combination of DSeA and TRAIL could be a novel strategy to overcome TRAIL resistance in malignant melanoma, and DSeA may be candidates for further evaluation as a chemosensitizer in clinical trails.

## INTRODUCTION

The increased incidence of malignant melanoma in the last decades, its high mortality and pronounced therapy resistance pose an enormous challenge [1]. Most advanced melanomas respond poorly to radiotherapy and chemotherapy and no effective therapy exists to inhibit the metastatic spread of this cancer [2]. Nowadays, most cancer therapies involve multiple agents, as it is almost universally the case that single drugs or single-target drugs is no longer appropriate for treatment of melanoma, owing to increasing dosage in the clinic has resulted in high toxicity, drug resistance, and unavoidable side effects [3]. Recently, combination chemotherapy has been found to be a superior treatment strategy that offers the potential for lowering the dose of chemotherapeutic drugs to reduce severe side effects.[4-7] Accumulative evidences suggested that the therapeutic targets for melanoma are the induction of apoptosis and suppression of survival pathways [1, 8]. Thus, it is badly needed to identify chemosensitizers to enhance the apoptotic inducing efficacy of clinical chemotherapeutic drugs and overcome multi-drug resistance (MDR) to kill malignant melanoma cells.

Tumor necrosis factor-related apoptosis-inducing ligand (TRAIL), as a member of the TNF family of proteins, has received a great deal of attention recently as novel therapeutic agents due to it induces apoptosis in a wide variety of transformed cells but not in normal cells *in vitro* and *in vivo* [9, 10]. Interaction of TRAIL with its specific receptors is capable of transducing apoptotic signal. Death receptors (DR4, DR5) are characterized by an intracellular death domain that facilitates assembly of the death-inducing signaling complexes (DISC) and subsequent activation of a caspase cascade, whereas the other three (TRAIL-R3, TRAIL-R4, and OPG) are decoy receptors, which possess dominant negative effects by competing with DR4 and DR5 for TRAIL interaction. On the other hand, Bid, a proapoptotic Bcl-2 family member, is also cleaved by caspase-8 or caspase-10 and then activates the mitochondrial apoptotic signaling pathway. Accordingly, the TRAIL-mediated death receptor pathway is considered to be an attractive candidate for cancer chemotherapy. Up to half of tumor cell lines, however, display resistance to TRAIL [11] and this resistance appears to be mediated through the regulation of cFLIP, Bcl-2 family members, IAP proteins, and activation of PI3K/Akt and extracellular signal-regulated kinases (ERK) survival pathway [12-14], which suggesting that treatment with TRAIL alone may be insufficient for cancer therapy. Therefore, agents are urgently needed that can sensitize the cancer cells to TRAIL. In this regard, a number of studies have shown the amplifying effect of anticancer drugs on TRAIL-mediated apoptosis via distinct signaling pathways [15-18].

Selenium (Se), an essential nonmetallic trace element, is a key component of several major metabolic pathways in human, including thyroid hormone metabolism, antioxidant defence system and immune function [19]. The role of selenocompounds as potential cancer chemopreventive and chemotherapeutic agents has been supported by epidemiological, preclinical and clinicalstudies [20]. Recent studies suggested that Selenocysteine (SeC), a nutritionally available selenoamino acid, exhibits potential applications in chemotherapy. In our previous works, SeC has been identified as a novel agent with stronger antiproliferative effect against human cancer cells through the induction of apoptosis, cell cycle arrest and also be able to synergize with chemo-therapeutic agents. For instance, SeC inhibits the growth of human melanoma cells in vivo and in vitro through induction of caspase-mediated apoptosis [21]. The combination of SeC and AF synergistically inhibited the growth of human breast cancer cells through induction of apoptosis by targeting TrxR [22]. Nevertheless, the poor solubility and stability limits the clinical application of SeC. Interestingly, 3, 3′-Diselenodipropionic acid (DSeA), a simple, stable, and water-soluble diselenide, possess similar structure with SeC, and has been reported for radioprotection, immuna-modulatory and anti-apoptosis [23, 24]. The molecular signaling involved in DSeA-mediated anti-cancer activity has never been investigated in any type of cancer cell lines. However, the previous results about the anticancer action of SeC prompted us to hypothesize that DSeA might have the potential to inhibit cancer cell growth or sensitize the cancer cells to chemotherapeutic drugs. Here, we report, for the first time, that DSeA synergistically enhances the apoptotic inducing efficacy of TRAIL in A375 cells but not in normal cells. The underlying molecular mechanisms through which they caused the cancer cell death were also elucidated. Taken together, our results demonstrate that, the combination of DSeA and TRAIL could be a novel strategy to overcome TRAIL resistance in malignant melanoma, and DSeA may be candidates for further evaluation as a chemosensitizer in clinical trails.

## RESULTS

### DSeA synergistically enhances the anticancer efficacy of TRAIL

**Figure 1:**
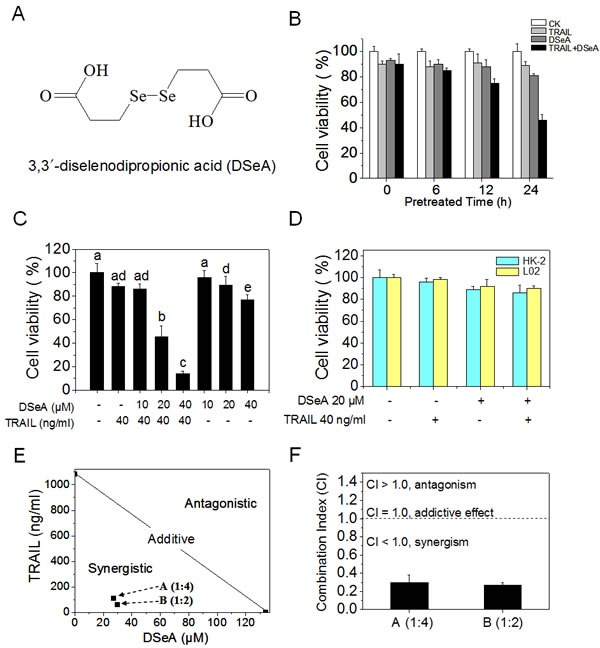
DSeA and TRAIL synergistically inhibit growth of A375 cells Cell viability was determined by MTT assay as described in Methods. (A) The chemical structure of DSeA. (B) DSeA enhances the efficacy of TRAIL-induced A375 cells growth inhibition. Cells were pretreated with or without 20 μM DSeA for 0, 6, 12 or 24 h and then incubated in the presence or absence of 40 ng/ml TRAIL for another 24 h. (C) Cells were pretreated with or without indicated concentrations of DSeA for 24 h and then co-treated with 40 ng/ml TRAIL for 24 h. (D) Cytotoxic effects of co-treatment on human normal cell lines HK-2 and L02. Cells were pretreated with 20 μM DSeA for 24 h and then expose to 40 ng/ml TRAIL for another 24 h. (E) Isobologram analysis of the synergistic antiproliferative effect of co-treatment on A375 cell. The data points (A, B) in the isobologram correspond to the actual IC_50_ value of DSeA and TRAIL with different ratio of concentrations in the combined treatment. DSeA (μM): TRAIL (ng/ml) = 1:4, 1:2. (F) The CI corresponding to different ratio of concentrations in the combined treatment. Each value represents the mean ± SD of three independent experiments. Bars with different characters (a, b, c, d and e) are statistically different at *p* < 0.05 level.

In the present study, human melanoma A375 cells, a TRAIL-resistance cell line was chose to evaluate the antiproliferative effects of combined DSeA (Fig. 1A) and TRAIL treatment by MTT assay. Firstly, the treatment of A375 cells with 10-320 μM DSeA for 6, 12, 24, 36 and 48 h or 10-1280 ng/ml TRAIL for 24 h inhibited cell proliferation in a time- and dose-dependent manner. In order to establish an optimal strategy in the combined treatment, cells were pretreated with different concentrations of DSeA for 0, 6, 12 and 24 h, and then co-treated with different concentrations of TRAIL for additional 24 h. As shown in Fig. 1B and Fig. 1C, pretreatment of cells with 20, 40 μM DSeA for 24 h and then co-treatment with 40 ng/ml TRAIL for 24 h significantly inhibits cell proliferation at 45.3% and 14.0%, respectively, indicating that the DSeA pretreatment notably enhances the growth inhibitory efficacy of TRAIL in a time- and dose-dependent manner. Despite the notable antiproliferative effects, the combined treatment with DSeA and TRAIL exhibited lower cytotoxicity towards human normal cell lines HK-2 and L02 (Fig. 1D).

To determine the interaction between DSeA and TRAIL, the growth inhibition of individual and combined treatments was evaluated by isobologram analysis. The growth inhibitory effect of combined DSeA and TRAIL treatment under different ratios (1:4 and 1:2) were found to be statistically synergistic, as evidenced by the location of the data points in the isobologram being far below from the line defining additive effect (Fig. 1E). In addition, the combination index (CI) of the co-treatments were calculated at 0.27 (1:2) and 0.30 (1:4) in Fig. 1F, which further confirmed the significant synergistic effects between DSeA and TRAIL. Therefore, DSeA could act as an efficient agent to overcome the TRAIL resistance in A375 cells.

### DSeA potentiates TRAIL-induced apoptosis by intrinsic and extrinsic apoptotic pathways

**Figure 2:**
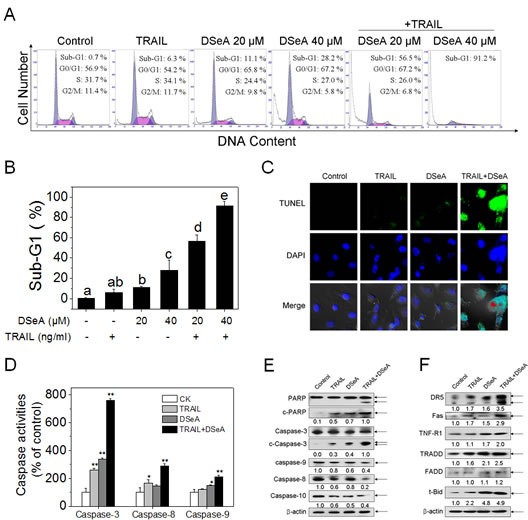
DSeA enhances TRAIL-induced A375 cells apoptosis though activation of mitochondrial and death receptor signaling pathways (A, B) Quantitative analysis of cell cycle distribution and apoptotic cell death induced by DSeA or/and TRAIL were measured by flow cytometric analysis. Cells were pretreated with or without 20, 40 μM DSeA for 24 h and then cultured in the presence or absence of 40 ng/ml TRAIL for another 24 h. Bars with different characters (a, b, c, d and e) are statistically different at *p* < 0.05 level. (C) Representative images of DNA fragmentation and nuclear condensation in response to 20 μM DSeA and/or 40 ng/ml TRAIL was examined by TUNEL and DAPI assay as described in Methods (magnification, 200×). All images shown are representative of three independent experiments with similar results. (D) Analysis of caspase activation induced by DSeA (20 μM), TRAIL (40 ng/ml) and co-treatment. Caspase activities were measured using synthetic fluorescent substrates for caspase-3/-8/-9. Each value represents the mean ± SD of three independent experiments, *, *p* < 0.05; **, *p* < 0.01 versus the control. (E, F) Western blot analysis of expression levels of PARP, caspase family members and death receptor signaling pathway-related proteins in A375 cells exposed to 20 μM DSeA and/or 40 ng/ml TRAIL. Changes in the levels of protein expression were shown as ratios of selected groups.

To elaborate the underlying mechanisms of synergistic antiproliferative effects induced by the combined treatment, cells after treatments were determined by flow cytometric analysis. As shown in Fig. 2A and Fig. 2B, exposure of A375 cells to DSeA and/or TRAIL resulted in marked increases in the proportion of apoptotic cells as reflected by the sub-diploid peaks. For instance, the sub-G1 population was slightly alternated after DSeA and TRAIL treatment alone, whereas noticeably potentiated from 0.7% to 56.5% and 91.2% after co-treatment with 20, 40 μM DSeA and 40 ng/ml TRAIL. However, no significant changes in cell cycle distribution were observed in the results (Fig. 2A). In order to further prove these findings, enzymatic labeling assay (TUNEL) and DAPI co-staining assay were carried out to detect DNA fragmentation and nuclear condensation. Results shown in Fig. 2C revealed a remarkably increase in DNA fragmentation and nuclear condensation in A375 cells induced by the co-treatment. These results demonstrate that the combined treatment-induced growth inhibition is mainly caused by induction of apoptosis. Moreover, as shown in Fig. 2D, the co-treatment induced noticeable activation of caspase-3, -8 and -9 in A375 cells, which demonstrate that both intrinsic and extrinsic apoptotic pathways were involved in co-treatment-induced apoptosis. To further confirm this hypothesis, Western blot analysis was used to examine activation of caspases and PARP, a biochemical marker of cells undergoing apoptosis. Results in Fig. 2E showed that the combined treatment effectively triggered the cleavage of PARP and activation of caspase-3, -9, -8 and -10. Furthermore, increase in expression levels of death receptor signaling pathways-related proteins, including death receptors (DR5, Fas and TNF-R1), adaptor protein (TRADD) and truncation of Bid (t-Bid), a death agonist member of BH3 domain-only protein family, were also observed in the combined treatment (Fig. 2F), which further confirmed the activation of death receptor-mediated extrinsic pathway in cell apoptosis.

### Induction of mitochondrial dysfunction by regulating the expression of Bcl-2 family members

**Figure 3:**
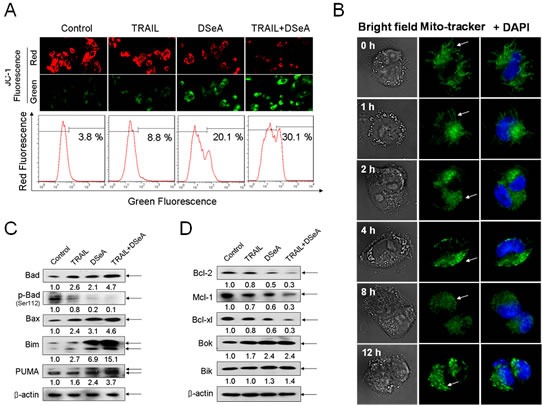
Effects of DSeA in combination with TRAIL on the function and structure of mitochondrial and expression levels of Bcl-2 family proteins in A375 cells Cells were pretreated with or without 20 μM DSeA for 24h and then co-treated with or without 40 ng/ml TRAIL for another 24 h. (A) After treatment cells were stained with JC-1 and detected by flow cytometric analysis and fluorescent microscope examination respectively. The depletion of mitochondrial membrane potential (*Δψ_m_*) was evidenced by the increment of the percentage of cells with green fluorescence. (B) Alternation of mitochondrial structure induced by DSeA in A375 cells. Cells were co-stained with fluorescent dye DAPI (nuclear) and mito-tracker (mitochondrial) in the dark at 37 °C, and then treated with 40 μM DSeA for the indicated time. After that cells were visualized under a fluorescent microscope (magnification, 1000×). All images shown are representative of three independent experiments with similar results. (C, D) Western blot analysis of the effects of DSeA in combination of TRAIL in Bcl-2 family member expression levels in A375 cells.

Mitochondria act as a point of integration for apoptotic signals originating from both the extrinsic and intrinsic apoptotic pathways. Loss of mitochondrial membrane potential (*Δψ_m_*) is associated with the activation of caspases and the initiation of apoptotic cascades. In this study, experiments were conducted to examine the status of mitochondria in DSeA- and/or TRAIL-treated A375 cells by flow cytometric analysis and fluorescent microscope examination using JC-1 as a molecular probe and mito-tracker as a marker of mitochondria. As shown in Fig. 3A, DSeA treatment and co-treatment-induced a marked elevation in depletion of *Δψ_m_*, as determined by the shift of fluorescence from red to green. The percentage of depolarized mitochondria increased from 3.8% to 30.1% in cells exposed to 20 μM of DSeA and 40 ng/ml of TRAIL. Furthermore, the results in Fig. 3B showed that healthy mitochondrial network (0 h) was extensively interconnected and appeared filamentous extended throughout the cytoplasm. The treatment of 40 μM DSeA resulted in obvious cytoplasmic shrinkage and mitochondrial fragmentation, which displayed a rapid onset after 1 h of treatment, followed by a progressive increase to 12 h. These evidences further confirmed the critical role of DSeA in co-treatment-induced the activation of mitochondrial-mediated apoptosis.

**Figure 4:**
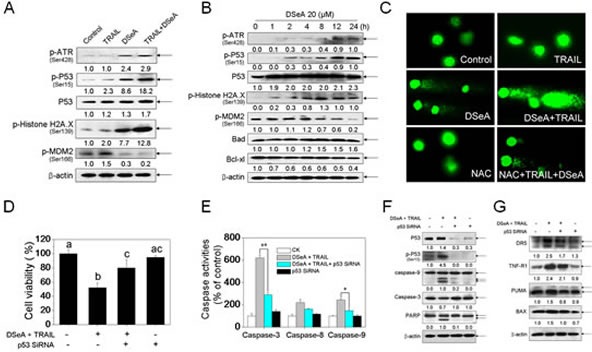
Co-treatment activated DNA damage-mediated p53-dependent apoptotic pathways in A375 cells (A) Western blot analysis of expression levels of phosphorylated ATR, p53, Histone H2A.X, MDM2 and Total p53 in A375 cells exposed to 20 μM DSeA for 24 h and/or 40 ng/TRAIL for another 24 h. (B) The time course of regulation of phosphorylated ATR, p53, Histone H2A.X, MDM2 and Total p53, Bcl-xl and Bax in A375 cells induced by DSeA. Cells were treated with 20 μM DSeA and harvested at various times. (C) Co-treatment-induced DNA damage in A375 cells. Cells after treatment were immediately analyzed by Comet assay as described in Methods. The length of tail reflects the degree of DNA damage in cells. (D, E) Protective effects of p53 siRNA on co-treatment-induced cell growth inhibition and caspases activation in A375 cells. Cells were pretreated with 50 nmol p53 siRNA for 24 h, and then treated in combination with DSeA and TRAIL. Cell viability was examined by MTT assay. Bars with different characters are statistically different at *p <* 0.05 level. Caspase activity was measured using synthetic fluorogenic substrate as described in Methods. Each value represents the mean ± SD of three independent experiments, *, *p* < 0.05; **, *p* < 0.01 versus the control. (F) Western blot analysis of the inhibitory effects of p53 siRNA on co-treatment-induced cell apoptosis, expression and activation of p53 and caspases.

Bcl-2 family members have been described as key regulators of mitochondrial permeability [25]. Therefore, we examined the effects of DSeA and/or TRAIL on the expression levels of pro-survival and pro-apoptotic Bcl-2 family proteins in A375 cells. As shown in Fig. 3C and Fig. 3D, Western blot analysis revealed that co-treatment significantly suppressed the expression of pro-survival Bcl-2 family proteins, such as Bcl-2, Mcl-1, and Bcl-xl, and up-regulated the expression of pro-apoptosis Bcl-2 family proteins, such as Bax, Bad, Bim and PUMA. Moreover, dephosphorylation of Bad at serine 112 was also observed in response to the combined treatment. The time course analysis showed that the alternation of the expression levels of Bcl-2 family proteins could be detected after treated with DSeA for 4 h (Fig. 4B). These results indicate that the combined treatment causes the depletion of *Δψ_m_* by regulating the expression of Bcl-2 family proteins.

### Activation of p53 pathway by triggering DNA damage

To confirm whether p53 signaling pathway is activated by the combined treatment, we determined the protein level of p53 and the related regulators in A375 cells after treated with indicated concentrations of DSeA and TRAIL. As shown in Fig. 4A, the treatment moderately increased the expression of total p53. A significant increase of phosphorylated p53 at serine 15 and phosphorylated ATR at serine 428 were detected in DSeA and combined treatment. Protein level of phosphorylated MDM2, a negative regulatory partner of p53, was also down-regulated in A375 cells treated with DSeA in combination with TRAIL. Results in Fig. 4B also showed that pretreatment of cells with 20 μM DSeA for 24 h notably alternated the status of cells, as evidenced by a rapid onset of ATR and p53 phosphorylation after 1-2 h of treatment, followed by a progressive elevation. We also showed that, DSeA and TRAIL significantly up-regulated the phosphorylation of histone H2A.X at serine 139 (Fig. 4A). The time course analysis revealed that significant increase in phosphorylated H2A.X was observed as early as 1 h of DSeA treatment, which was prior to phosphorylation of p53 (Fig. 4B). Furthermore, as shown in Fig. 4C, the combined treatment induced a remarkable DNA damage, as evidenced by the increase in tail DNA in A375 cells. These results suggest that, DSeA and TRAIL activate p53 pathway by induction of DNA damage.

To further examine the role of p53 in cell apoptosis, we used p53 siRNA to reduce the mRNA expression level of this gene and examined its effects on cell viability, caspases activities and the expression of PIGs. As shown in Fig. 4F, transfection with p53 siRNA significantly down-regulated the expression levels of total and phosphorylated p53 and effectively blocked the co-treatment-induced cell growth inhibition (Fig. 4D), activation of caspase-3, caspase-8 and caspase-9 (Fig. 4E, 4F) and PARP cleavage (Fig. 4F). Results in Fig. 4G showed that the expression levels of PIGs, including DR5, PUMA and Bax in the combined treatment were decreased by the transfection, whereas no obvious change in expression level of TNF-R1 was observed. In addition, the control siRNA showed no effects on co-treatment-induced p53 activation and apoptosis. Taken together, these data indicate that the activation of p53 signaling pathway is required for co-treatment-induced apoptosis.

### PI3K/Akt but not MAPKs contributes to cell apoptosis

Studies have shown that, upon stress stimuli, MAPKs phosphorylate and activate p53, leading to p53-mediated cellar response [26, 27]. Therefore, in this study, we examined whether the MAPKs were activated in DSeA- and/or TRAIL-treated A375 cells by Western blotting. It was found that co-treatment moderately increased the expression levels of phosphorylated and total JNK in A375 cells, but showed no effects on p38 and ERK (Fig. S1A). Moreover, their specific inhibitors reduced their phosphorylation levels, but exhibited no obvious effect on co-treatment-induced cell growth inhibition (Fig. S1B), demonstrating that co-treatment-induced cell apoptosis is independent of MAPKs activation.

**Figure 5:**
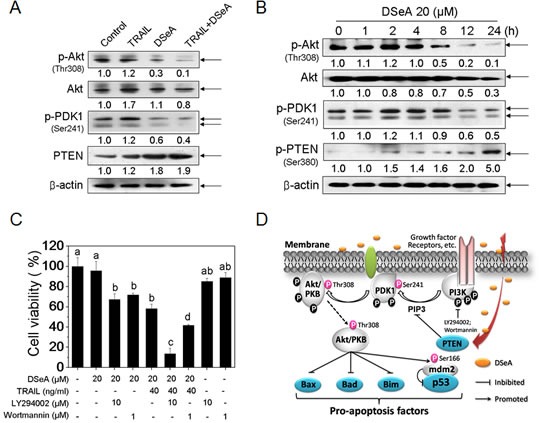
Critical Roles of PI3K/Akt Signaling pathway in A375 cells growth inhibition induced by co-treatment (A) Effects of DSeA and/or TRAIL on the expression levels of phosphorylated Akt, PDK1, and total Akt and PTEN. Cells were preatreated with 20 μM DSeA for 24 h and then co-treated with 40 ng/ml TRAIL for another 24 h. (B) Western blot analysis of time-dependent regulation of Akt in A375 cells. Cells were treated with 20 μM DSeA for various periods of time. (C) Effects of LY294002 and Wortmannin (PI3K inhibitor) on co-treatment-induced A375 cells growth inhibition. Cells were pretreated with 10 μM LY294002 or 1μM Wortmannin for 1 h prior to the co-treatment. Cell viability was determined by MTT assay. All data are expressed as means ± SD of triplicates. Bars with different characters (a, b and c) are statistically different at *p* < 0.05 level. (D) Proposed inhibition of pro-survival PI3K/Akt signaling pathway triggered by DSeA in A375 cells. Growth factor receptor–ligand interactions recruit and activate PI3K, which modulates PIP3 generation. PIP3 binds to AKT, allowing its translocation to the plasma membrane and then phosphorylated by PDK1 at the sites of Thr308. The activated Akt promotes cell survival by phosphorylating and inactivating several targets, including Bad, Bax, Bim and MDM2. PTEN, as a major negative regulator (PIP3 inhibitor) in PI3K/Akt pathway, is increased by co-treatment.

PI3K/Akt pathway has been found closely related to chemo-resistance in many tumors [28]. Herein we showed that, the expression levels of phosphorylated Akt and PDK1 were slightly up-regulated by TRAIL treatment, whereas significantly down-regulated by DSeA or co-treatment (Fig. 5A). Furthermore, a moderate increase in PTEN expression was observed in the combined treatment. Results in Fig. S2 showed that the expression levels of PTEN in the combined treatment were decreased by p53 siRNA transfection. A time course study showed that Akt and PKD1 phosphorylation increased rapidly after1 h of DSeA treatment, peaked at approximately 2 h, progressively declined from 8 to 24 h (Fig. 5B), which could be associated with the enhancement of PTEN expression. Unexpectedly, an obvious phosphorylation of PTEN at the site of serine 380 was also observed in DSeA-treated cells (Fig. 5B), which means PTEN, may not be the critical factor of DSeA-mediated Akt dephosphorylation. To further confirm our hypothesis, the antiproliferative activities of co-treatment against a panel of three PTEN-mutant human cancer cell lines and a PTEN-wild type cell line were examined by MTT assay. Results in Fig. S3 showed that DSeA synergistically enhanced the anticancer efficacy of TRAIL in MDA-MB-468 (PTEN-mutant) cell line, whereas no obvious synergistic effects were observed in other cell lines. These results demonstrate that DSeA potentiates TRAIL-induced cell growth inhibition in a PTEN-independent manner, which means PTEN, may not be the critical factor of DSeA-mediated Akt dephosphorylation and cell death in human melanoma A375 cells.

In addition, PI3K inhibitor LY294002 and wortmannin were used to evaluate whether suppression of PI3K/Akt pathway was needed in co-treatment-induced cell growth inhibition. As shown in Fig. 5C, treatment with 10 μM LY294002, 1 μM Wortmannin or 20 μM DSeA alone for 24 h showed no significant inhibitory effects on the growth of A375 cells. However, pretreatment with LY294002 or Wortmannin for 1 h significantly increased the proportion of cell death induced by DSeA or combined treatment. These results demonstrate that inactivation of PI3K/Akt signaling pathway was involved in co-treatment-induced cell death in A375 cells.

### Essential role of oxidative stress in co-treatment-induced apoptosis and the related cellular events in A375 cells

**Figure 6:**
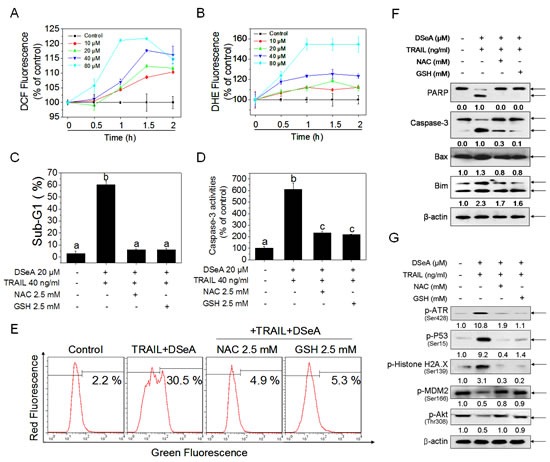
Antioxidants protect A375 cells from co-treatment-induced apoptosis by blocking Akt and MDM2 dephosphorylation, DNA damage-mediated p53 activation and mitochondrial dysfunction (A, B) DSeA-induced accumulation of ROS in A375 cells. Cells were treated with indicated concentrations of DSeA for various periods of time. The levels of intracellular ROS were determined by DCF and DHE assay. (C, D) Inhibitory effects of NAC and GSH on co-treatment-induced accumulation of sub-G1 cell population and activation of caspase-3. Cells were pretreated with or without 2.5 mM NAC or GSH for 1 h, and then co-treated with 20 μM DSeA for 24h, followed by treated with 40 ng/ml TRAIL for another 24 h. Quantitative analysis of cell cycle distribution and apoptotic cell death were measured by flow cytometric analysis. Caspase activities were measured using synthetic fluorescent substrates for caspase-3. Bars with different characters (a, b and c) are statistically different at p < 0.05 level. (E) Protective effects of NAC and GSH on co-treatment-induced the depletion of Δψm in A375 cells. Cells after treatment were stained with JC-1 and detected by flow cytometric analysis. (F, G) Western blot analysis of the effects of NAC and GSH on co-treatment-induced the alternation of apoptosis-related protein expression levels.

It has been reported that reactive oxygen species (ROS) play an important role in the induction of apoptosis by Se compounds [29, 30]. Excess ROS is able to activate a variety of stress responses through activation or inhibition of distinct kinases [31]. As shown in Fig. 6A and Fig. 6B, DSeA treatments triggered a time- and dose-dependent increase in DCF and DHE fluorescence intensity, which indicates that up-regulation of intracellular ROS and superoxide radicals levels are early event in DSeA-induced apoptosis. Moreover, pretreatment of cells with 2.5 mM NAC or GSH completely blocked DSeA-induced apoptotic cell death in A375 cells, as reflected by sub-G1 peaks down-regulation, caspase-3 activation and PARP cleavage (Fig. S4). Results in Fig.6C, D and S5 also showed the significant inhibitory effect of antioxidants on cell apoptosis. Furthermore, as shown in Fig. 6E and Fig. 6F, pretreatment with NAC or GSH effectively inhibited the depletion of *Δψ_m_* though down-regulation of pro-apoptotic Bcl-2 family members Bim and Bax. Results in Fig. 4C and Fig. 6G showed that, activation of DNA damage-mediated ATR, p53 and H2A.X phosphoryaltion and phosphorylation of PTEN (Fig. S6) were also significantly reversed by the pretreatment with antioxidants. Protein levels of phosphorylated Akt and its downstream effecter MDM2 were also up-regulated by NAC and GSH (Fig. 6G), which suggests that ROS may act as an upstream mediator for PTEN-mediated Akt inactivation.

## DISCUSSION

TRAIL has received a great deal of attention as novel therapeutic agents due to their high potential for selective killing activity against transformed cells [9, 10]. However, over 50% tumor cells, including malignant melanoma [11], are intrinsically or acquired resistant to TRAIL treatment alone. Therefore, it is of great importance to search for agents that could enhance the sensitivity of cancer cells to TRAIL. In this study, we showed for the first time that DSeA, as a potential chemosensitizer, synergistically enhanced the apoptotic inducing efficacy of TRAIL in A375 cells but not in normal cells. Results of mechanistic studies indicated that DSeA significantly potentiates TRAIL-induced apoptosis in A375 cells by triggering ROS-dependent DNA damage, p53 phosphorylation and PI3K/Akt signaling pathway inactivation.

As a result of the balance between therapeutic potential and toxic side effects of a strategy is very important when evaluating its usefulness for cancer treatment, experiments were designed to investigate the *in vitro* cytotoxicity of DSeA and/or TRAIL against human melanoma A375 cell and normal cell lines (HK-2, L02). Based on the results obtained from MTT assay (Fig. 1), DSeA was identified as a novel chemosensitizer with notable efficience to synergy with TRAIL to inhibit A375 cell proliferation. On the contrary, co-treatment was found to show lower cytotoxicity towards normal human cells, which suggests that the strategy of DSeA in combination with TRAIL possesses great selectivity between cancer and normal cells and displays application potential in cancer chemoprevention and chemotherapy. Studies have suggested that many antitumor agents used in chemotherapy usually cause apoptosis or cell cycle arrest at the G0/G1 or G2/M phases on cancer cells [32]. Herein, we showed that, exposure of A375 cells to DSeA and TRAIL resulted in marked accumulation of sub-G1 cell population (Fig. 2A, 2B), DNA fragmentation and nuclear condensation (Fig. 2C), caspase-3 activation and PARP cleavage (Fig. 2D, 2E), whereas no significant changes in cell cycle distribution were observed. These results indicate that apoptosis is the major mode of cell death triggered by the combined treatment.

Generally, apoptosis can be initiated by two central mechanisms, the death receptor mediated-extrinsic and mitochondrial mediated-intrinsic pathways. In the present study, we provided convincing evidence that DSeA significantly enhanced TRAIL-induced apoptosis though activation of the extrinsic pathway (Fig. 2F). Furthermore, the extrinsic pathway was also found to crosstalk with the intrinsic pathway through the truncation of Bid, which relayed the apoptotic signal from the cell surface to mitochondria. Considerable evidence suggests that selenocompounds potentiated the effect of TRAIL-induced apoptosis in cancer cells through activation of mitochondrial pathway [33, 34]. In this study, treatment of DSeA alone or in combination with TRAIL resulted in significant depletion of *Δψ_m_*, mitochondrial fragmentation and release of mitochondrial contents through regulation of Bcl-2 family members.

Tumor suppressor gene p53 has been reported to be able to directly or indirectly induce cell apoptosis through both the extrinsic and intrinsic apoptosis pathways [35]. Expression of a host of apoptotic genes is stimulated by p53, particularly those involved in the mitochondrial apoptotic pathway such as Bax, NOXA, PUMA and APAF1. In addition, p53 stimulates expression of genes involved in the death receptor pathway including DR5, FAS and PIDD [36]. Herein, our results demonstrated that p53 was an important regulator in co-treatment-induced A375 cell apoptosis (Fig. 4). Phosphorylation of p53 has been reported to play a major role in cellular response to DNA damage, which leads to reduced interaction between p53 and its negative regulator, oncoprotein MDM2 [37]. Phosphorylation of MDM2 could block its binding to p19ARF, increasing the degradation of p53 [38]. Thus, the dephosporylation of MDM2 contributed to the stability of p53 in A375 cell apoptosis (Fig. 4A). Studies have reported that DNA damage can activate the p53 pathway by activating various damage sensor proteins such as ATM, ATR, DNA-PK, and protein kinases like Chk1 and Chk2 [39]. We have previously showed that, selenocompounds could induce cancer cell apoptosis though triggering DNA damage-mediated p53 phosphorylation. [21, 22, 30] Herein we showed that, DNA damage was as an early cellular event during cell apoptosis, which subsequently activated the p53 pathway (Fig. 4C). Moreover, silencing of p53 effectively down-regulated the expression levels of total p53, Ser 15-p53 and PIGs, and suppressed caspases activation, which suggest that cell apoptosis induced by DSeA and TRAIL is p53 dependent.

In addition to triggering a proapoptotic signal through activation of death receptor pathway, TRAIL treatment can also activate diverse intracellular signaling pathways, including NF-κB, PI3K and MAPKs that can stimulate cell survival and proliferation. Many studies have reported the regulation of MAPK pathways as likely mechanisms for induction of apoptosis in cancer cells by these Se compounds [40, 41]. However, our results suggested that MAPKs did not play important roles in regulating the cell apoptosis. The PI3K/Akt pathway regulates fundamental cellular functions such as cell growth, survival, and movement [42, 43]. Negative regulation of the PI3K/Akt pathway is mainly accomplished by the dual function lipid and protein phosphatase PTEN, which dephosphorylates the D3 position of PIP2 and PIP3, thereby counteracting the activity of PI3K [28]. Overexpression of PTEN uniformly inhibited colony formation, implicating a tumor-suppressive function of PTEN in melanoma[44]. A number of factors, including p53 have been shown to transcriptionally regulate PTEN mRNA[45]. In the present study, DSeA or co-treatment moderately increased the expression level of PTEN which down-regulated by the transfection of p53 siRNA. These data suggest that the dephosphorylation of Akt by DSeA or co-treatment could be associated with p53-dependent PTEN overexprsssion.

The activated Akt mediates cell growth via the phosphorylation of many substrates including Bax [46], Bad [47], MDM2 [48] and FOXO transcription factors, which can regulate the expression of Bim [49]. For instance, Akt-mediated phosphorylation of two residues, ser166 and ser186 in MDM2, has been reported to stimulate nuclear import of MDM2 and MDM2-mediated p53 degradation [48]. Under these circumstances, p53 induction becomes acutely sensitive to DNA damage-inducing drugs [50]. Accumulative evidences have showed that several cancer cells were resistant to TRAIL due to high constitutively active Akt [12, 13, 51]. Downregulation of Akt by the PI3K inhibitors reversed the TRAIL resistance, while transfecting constitutively active Akt into cells with low Akt activity attenuated TRAIL-induced apoptosis [12, 13, 51, 52]. Recent studies suggest that the regulation of the PI3K/Akt pathways, as likely mechanisms, were involved in Se compounds alone or in combination with chemotherapeutic drugs-induced apoptosis in human cancer cells [22, 41, 53, 54]. In this study, we have established a mechanistic link between the PI3K/Akt pathway and cell apoptosis induced by DSeA and TRAIL. PI3K/Akt pathway plays important roles in regulating cell death induced by TRAIL, while DSeA was sufficient to overcome PI3K/Akt pathway-mediated TRAIL resistance in A375 cells by enhancement of PTEN expression (Fig. 5).

As a critical upstream initiator in intracellular signaling cascades, ROS can control cell growth, proliferation, migration, and apoptosis though regulation of the activity of certain enzymes [55]. Growing evidence has suggested that accumulation of ROS acted as an important cellular event induced by selenocompounds in cancer cells [21, 29, 30, 56]. In our recent studies, SeC was identified as a pro-oxidant selenocompound that could induce cancer cell apoptosis by the activation of oxidative stress [22]. In this study, we showed that, overproduction of ROS was involved in cell apoptosis induced by DSeA, as a Selenocysteine derivative. Excessive production of ROS could attack various components of DNA, leading to generation of a variety of ROS-mediated modified products, including oxidized bases, DNA strand breaks, DNA intra-strand adducts, and DNA-protein crosslinks [57]. In this study, as shown in Fig. 4C and Fig. 6, antioxidants significantly blocked the DNA damage, p53 activation and depletion of *Δψ_m_* induced by DSeA and TRAIL. Besides the induction of oxidative damage, overproduction of ROS was also able to activate PI3K/Akt signaling pathway, which is the major oxidative stress-sensitive signal transduction pathways in most cell types [31]. Interestingly, we also found that, NAC and GSH significantly inhibited the combined treatment-induced dephosphorylation of Akt and its downstream effecter MDM2 (Fig. 6F). Taken together, ROS acts as an upstream regulator of Akt and p53 signaling pathways.

**Figure 7:**
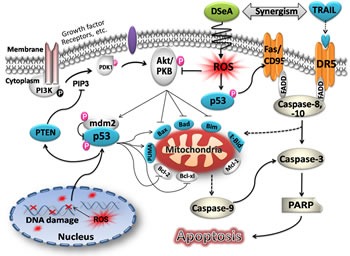
Proposed apoptosis-inducing signaling pathway triggered by DSeA and TRAIL in A375 cells DSeA first indues intracellular ROS overproduction in a rapid time. The accumulation of intracellular ROS induces DNA damage-mediated p53 activation and dephosphorlation of Akt, which is negative regulated by PTEN. Inactivation of Akt and phosphorylation of p53 co-regulate the expression of Bcl-2 family proteins, resulting in depletion of *Δψ_m_* and release of apoptogenic factors from the mitochondria into cytosol. Furthermore, the activation of p53 increases the expression of death receptors, which accompany with mitochondrial pathway to promote TRAIL-induced apoptosis through activation of caspase cascade and PARP cleavage.

In summary, our data provides compelling evidence that DSeA synergistically enhances the efficacy of TRAIL-induced apoptosis in A375 cells by triggering ROS overproduction, which activated DNA damage-mediated p53 phosphorylation and suppressed PI3K/Akt-mediated resistance (Fig. 7). Our results suggest that the combination of DSeA and TRAIL could be a novel strategy to overcome PI3K/Akt signaling pathway-mediated resistance in malignant melanoma cells and DSeA may be candidates for further evaluation as a chemosensitizer in clinical trails.

## MATERIALS AND METHODS

### Reagents

Thiazolyl blue tetrazolium bromide (MTT), propidium iodide (PI), 5,5′,6,6′- tetrachloro-1,1′,3,3′-tetraethylbenzimidazolcarbocyanine iodine (JC-1), 4′, 6-diamidino-2-phenyindole (DAPI), N-acetylcysteine (NAC), glutathione (GSH), 2′,7′-dichlorofluorescein diacetate (DCF-DA) and 3,3′-diselenodipropionic acid (DSeA) were purchased from Sigma-Aldrich. Recombinant human TRAIL/APO2L was obtained from Merck Millipore (Billerica, MA). The mitochondrial dye (mito-tracker) green was purchased from Invitrogen, Molecular Probes (Eugene, OR, USA). Caspase-3 substrate (Ac-DEVD-AMC), caspase-8 substrate (IETD-AFC) and Caspase-9 substrate (Ac-LEHD-AFC) were obtained from Calbiochem (San Diego, CA). Bicinchoninic acid (BCA) kit for protein determination and superoxide radicals probe Dihydroethidium (DHE) were purchased from Beyotime (Haimen, China). X-tremeGene siRNA transfection reagent was obtained from Roche Applied Science (Mannheim, Germany). P53 small interfering RNAs (siRNA), SP600125, SB203580, U0126, LY294002, Wortmannin and all the antibodies used in this study were purchased from Cell Signaling Technology (Beverly, MA). The water used in cellular experiments was ultrapure, supplied by a Milli-Q water purification system from Millipore.

### Cell culture

The cell lines used in this study, including human melanoma A375 cells human glioblastoma U87, U251 cells, human breast adrenocarcinoma MCF-7 cells and normal human kidney HK-2 cells, were obtained from American Type Culture Collection (ATCC, Manassas, VA). Human normal liver cells L02 and triple negative breast cancer cells MDA-MB-468 were purchased from kyGEN (Nanjing, China). The cell lines were maintained in DMEM medium supplemented with fetal bovine serum (10%), penicillin (100 units/mL), and streptomycin (50 units/mL) at 37 °C in a humidified incubator with 5% CO_2_ atmosphere.

### Determination of cell viability

The effects of DSeA and/or TRAIL on the cell proliferation were determined by MTT and trypan blue staining assays [58].

### Isobologram analysis

The synergistic effect between DSeA and TRAIL was evaluated by the isobologram method [59]. Briefly, a straight line was formed by plotting the IC_50_ values of DSeA and TRAIL on the x- and y-axes, respectively. The data point in the isobologram corresponds to the actual IC_50_ value of DSeA and TRAIL with different ratio of concentrations in the combined treatment. If a data point is on or near the line, which represents additive effect, whereas a data point that lies below or above the line indicates synergism or antagonism, respectively. Further more, the combination index (CI) was also calculated to examine the interaction between DSeA and TRAIL. CI value: < 1.0, synergism; = 1.0, additive effect; > 1.0, antagonism.

### Determination of cell apoptosis

The effects of DSeA and/or TRAIL on the cell cycle progression and the induction of apoptotic cell death were quantified by flow cytometric analysis according to our previous method [58]. DNA fragmentation was examined by the TUNEL apoptosis detection kit (Roche) following the manufacturer's instruction. Caspase activity was determined by fluorescence assay using specific substrates [41].

### Evaluation of mitochondrial membrane potential (Δψm) and structure

Cells after treatments were harvested and resuspended in PBS buffer containing 10 μg/ml of JC-1. The cells were incubated at 37 °C in the incubator for 10 min, and then the staining solution was removed. The cells were washed with PBS and analyzed by flow cytometer or visualized under a fluorescence microscope (Life technologies EVOS® FL Auto). The percentage of the green fluoresce from JC-1 monomers was used to represent the cells that lost Δψm.

Alternation of mitochondrial structure was detected by mito-tracker (green) and DAPI (blue) co-staining, and visualized under a fluorescent microscope (Life technologies EVOS® FL Auto, 1000×).

### Measurement of ROS generation and DNA damage

The effects of DSeA on accumulation of superoxide radicals in A375 cells were evaluated by DCF and DHE fluorescence assay. Single cell gel electrophoresis (Comet assay) was used to detect DSeA –induced DNA damage [21].

### RNA interference

The tansfection of p53 siRNA into A375 cells was carried out according to the previous method [21].

### Western blotting

The cells were harvested and incubated with cell lysis buffer overnight at −20°C. Protein electrophoresis and blotting was done according to the previous methods [58]. After then, the membranes were washed with TBST buffer and incubated with antibodies. The target proteins were detected on X-ray film (Kodak) using an enhanced chemiluminescence reagent. β-actin was used to confirm the equal loading and transfer of proteins.

### Statistics analysis

Experiments were carried out at least in triplicate and results were expressed as mean ± SD. Statistical analysis was performed using SPSS statistical program version 13 (SPSS Inc., Chicago, IL). Difference between two groups was analyzed by two-tailed Student's t test and that between three or more groups was analyzed by one-way ANOVA multiple comparisons. Difference with *p* < 0.05 (*) or *p* < 0.01 (**) was considered statistically significant.

## SUPPLEMENTARY MATERIAL FIGURES



## References

[1] Eberle J, Kurbanov BM, Hossini AM, Trefter U, Fecker LF (2007). Overcoming apoptosis deficiency of melanoma - Hope for new therapeutic approaches. Drug Resist Update.

[2] Gogas HJ, Kirkwood JM, Sondak VK (2007). Chemotherapy for metastatic melanoma - Time for a change?. Cancer.

[3] Perlis C, Herlyn M (2004). Recent advances in melanoma biology. Oncologist.

[4] Fan C, Zheng W, Fu X, Li X, Wong Y-S, Chen T (2014). Strategy to enhance the therapeutic effect of doxorubicin in human hepatocellular carcinoma by selenocystine, a synergistic agent that regulates the ROS-mediated signaling. Oncotarget.

[5] Chiarini F, Lonetti A, Teti G, Orsini E, Bressanin D, Cappellini A, Ricci F, Tazzari PL, Ognibene A, Falconi M, Pagliaro P, Iacobucci I, Martinelli G, Amadori S, McCubrey JA, Martelli AM (2012). A combination of temsirolimus, an allosteric mTOR inhibitor, with clofarabine as a new therapeutic option for patients with acute myeloid leukemia. Oncotarget.

[6] Schmukler E, Wolfson E, Haklai R, Elad-Sfadia G, Kloog Y, Pinkas-Kramarski R (2013). Chloroquine synergizes with FTS to enhance cell growth inhibition and cell death. Oncotarget.

[7] Lin Y-C, Wu M-H, Wei T-T, Lin Y-C, Huang W-C, Huang L-Y, Lin Y-T, Chen C-C (2013). Metformin sensitizes anticancer effect of dasatinib in head and neck squamous cell carcinoma cells through AMPK-dependent ER stress. Oncotarget.

[8] Shtivelman E, Davies MA, Hwu P, Yang J, Lotem M, Oren M, Flaherty KT, Fisher DE (2014). Pathways and therapeutic targets in melanoma. Oncotarget.

[9] Hall MA, Cleveland JL (2007). Clearing the TRAIL for cancer therapy. Cancer Cell.

[10] Ashkenazi A (2002). Targeting death and decoy receptors of the tumour-necrosis factor superfamily. Nat Rev Cancer.

[11] Walczak H, Miller RE, Ariail K, Gliniak B, Griffith TS, Kubin M, Chin W, Jones J, Woodward A, Le T, Smith C, Smolak P, Goodwin RG, Rauch CT, Schuh JC, Lynch DH (1999). Tumoricidal activity of tumor necrosis factor-related apoptosis-inducing ligand in vivo. Nat Med.

[12] Chen XF, Thakkar H, Tyan F, Gim S, Robinson H, Lee C, Pandey SK, Nwokorie C, Onwudiwe N, Srivastava RK (2001). Constitutively active Akt is an important regulator of TRAIL sensitivity in prostate cancer. Oncogene.

[13] Nesterov A, Lu XJ, Johnson M, Miller GJ, Ivashchenko Y, Kraft AS (2001). Elevated Akt activity protects the prostate cancer cell line LNCaP from TRAIL-induced apoptosis. J Bio Chem.

[14] Kim Y-H, Lee D-H, Jeong J-H, Guo ZS, Lee YJ (2008). Quercetin augments TRAIL-induced apoptotic death: Involvement of the ERK signal transduction pathway. Biochem Pharmacol.

[15] Chen K-F, Tai W-T, Liu T-H, Huang H-P, Lin Y-C, Shiau C-W, Li P-K, Chen P-J, Cheng A-L (2010). Sorafenib Overcomes TRAIL Resistance of Hepatocellular Carcinoma Cells through the Inhibition of STAT3. Clin Cancer Res.

[16] Prasad S, Yadav VR, Ravindran J, Aggarwal BB (2011). ROS and CHOP Are Critical for Dibenzylideneacetone to Sensitize Tumor Cells to TRAIL through Induction of Death Receptors and Downregulation of Cell Survival Proteins. Cancer Res.

[17] Saturno G, Valenti M, De Haven Brandon A, Thomas GV, Eccles S, Clarke PA, Workman P (2013). Combining trail with PI3 kinase or HSP90 inhibitors enhances apoptosis in colorectal cancer cells via suppression of survival signaling. Oncotarget.

[18] Fulda S (2011). Novel insights into the synergistic interaction of Bortezomib and TRAIL: tBid provides the link. Oncotarget.

[19] Rayman MP (2000). The importance of selenium to human health. The Lancet.

[20] Sinha R, Ei-Bayoumy K (2004). Apoptosis is a critical cellular event in cancer chemoprevention and chemotherapy by selenium compounds. Curr Cancer Drug Tar.

[21] Chen T, Wong YS (2008). Selenocystine induces apoptosis of A375 human melanoma cells by activating ROS-mediated mitochondrial pathway and p53 phosphorylation. Cell Mol Life Sci.

[22] Liu C, Liu Z, Li M, Li X, Wong Y-S, Ngai S-M, Zheng W, Zhang Y, Chen T (2013). Enhancement of Auranofin-Induced Apoptosis in MCF-7 Human Breast Cells by Selenocystine, a Synergistic Inhibitor of Thioredoxin Reductase. Plos One.

[23] Kunwar A, Bag PP, Chattopadhyay S, Jain VK, Priyadarsini KI (2011). Anti-apoptotic, anti-inflammatory, and immunomodulatory activities of 3,3 ‘-diselenodipropionic acid in mice exposed to whole body gamma-radiation. Arch Toxicol.

[24] Kunwar A, Bansal P, Kumar SJ, Bag PP, Paul P, Reddy ND, Kumbhare LB, Jain VK, Chaubey RC, Unnikrishnan MK, Priyadarsini KL (2010). In vivo radioprotection studies of 3,3 ‘-diselenodipropionic acid, a selenocystine derivative. Free Radical Bio Med.

[25] Cory S, Adams JM (2002). The Bcl2 family: regulators of the cellular life-or-death switch. Nat Rev Cancer.

[26] Fan MY, Chambers TC (2001). Role of mitogen-activated protein kinases in the response of tumor cells to chemotherapy. Drug Resist Update.

[27] Chappell WH, Steelman LS, Long JM, Kempf RC, Abrams SL, Franklin RA, Baesecke J, Stivala F, Donia M, Fagone P, Malaponte G, Mazzarino MC, Nicoletti F, Libra M, Maksimovic-Ivanic D, Mijatovic S (2011). Ras/Raf/MEK/ERK and PI3K/PTEN/Akt/mTOR Inhibitors: Rationale and Importance to Inhibiting These Pathways in Human Health. Oncotarget.

[28] West KA, Castillo SS, Dennis PA (2002). Activation of the PI3K/Akt pathway and chemotherapeutic resistance. Drug Resist Update.

[29] Zhao R, Xiang N, Domann FE, Zhong WX (2006). Expression of p53 enhances selenite-induced superoxide production and apoptosis in human prostate cancer cells. Cancer Res.

[30] Chen T, Wong Y-S (2009). Selenocystine induces caspase-independent apoptosis in MCF-7 human breast carcinoma cells with involvement of p53 phosphorylation and reactive oxygen species generation. Int J Biochem Cell B.

[31] Pelicano H, Carney D, Huang P (2004). ROS stress in cancer cells and therapeutic implications. Drug Resist Updat.

[32] Kim R (2005). Recent advances in understanding the cell death pathways activated by anticancer therapy. Cancer.

[33] Hu H, Jiang C, Schuster T, Li G-X, Daniel PT, Lu J (2006). Inorganic selenium sensitizes prostate cancer cells to TRAIL-induced apoptosis through superoxide/p53/Bax-mediated activation of mitochondrial pathway. Mol Cancer Ther.

[34] Yamaguchi K, Uzzo RG, Pimkina J, Makhov P, Golovine K, Crispen P, Kolenko VM (2005). Methylseleninic acid sensitizes prostate cancer cells to TRAIL-mediated apoptosis. Oncogene.

[35] Hofseth LJ, Hussain SP, Harris CC (2004). p53: 25 years after its discovery. Trends in Pharmacol Sci.

[36] Vousden KH, Lu X (2002). Live or let die: The cell's response to p53. Nat Rev Cancer.

[37] Shieh SY, Ikeda M, Taya Y, Prives C (1997). DNA damage-induced phosphorylation of p53 alleviates inhibition by MDM2. Cell.

[38] Zhou BHP, Liao Y, Xia WY, Zou YY, Spohn B, Hung MC (2001). HER-2/neu induces p53 ubiquitination via Akt-mediated MDM2 phosphorylation. Nat Cell Bio.

[39] Sancar A, Lindsey-Boltz LA, Unsal-Kacmaz K, Linn S (2004). Molecular mechanisms of mammalian DNA repair and the DNA damage checkpoints. Annu Rev Biochem.

[40] Goel A, Fuerst F, Hotchkiss E, Boland R, Boland CR (2006). Selenomethionine induces p53 mediated cell cycle arrest and apoptosis in human colon cancer cells. Cancer Bio Ther.

[41] Chen T, Wong Y-S (2008). Selenocystine Induces S-Phase Arrest and Apoptosis in Human Breast Adenocarcinoma MCF-7 Cells by Modulating ERK and Akt Phosphorylation. J Agr and Food Chem.

[42] Datta SR, Brunet A, Greenberg ME (1999). Cellular survival: a play in three Akts. Genes Dev.

[43] McCubrey JA, Steelman LS, Chappell WH, Abrams SL, Montalto G, Cervello M, Nicoletti F, Fagone P, Malaponte G, Mazzarino MC, Candido S, Libra M, Baesecke J, Mijatovic S, Maksimovic-Ivanic D, Milella M (2012). Mutations and Deregulation of Ras/Raf/MEK/ERK and PI3K/PTEN/Akt/mTOR Cascades Which Alter Therapy Response. Oncotarget.

[44] Tsao H, Zhang X, Fowlkes K, Haluska FG (2000). Relative reciprocity of NRAS and PTEN/MMAC1 alterations in cutaneous melanoma cell lines. Cancer Res.

[45] Stambolic V, MacPherson D, Sas D, Lin Y, Snow B, Jang Y, Benchimol S, Mak TW (2001). Regulation of PTEN transcription by p53. Mol Cell.

[46] Gardai SJ, Hildeman DA, Frankel SK, Whitlock BB, Frasch SC, Borregaard N, Marrack P, Bratton DL, Henson PM (2004). Phosphorylation of Bax Ser(184) by Akt regulates its activity and apoptosis in neutrophils. J Bio Chem.

[47] Datta SR, Dudek H, Tao X, Masters S, Fu H, Gotoh Y, Greenberg ME (1997). Akt phosphorylation of BAD couples survival signals to the cell-intrinsic death machinery. Cell.

[48] Ogawara Y, Kishishita S, Obata T, Isazawa Y, Suzuki T, Tanaka K, Masuyama N, Gotoh Y (2002). Akt enhances Mdm2-mediated ubiquitination and degradation of p53. J Bio Chem.

[49] Manning BD, Cantley LC (2007). AKT/PKB signaling: Navigating downstream. Cell.

[50] Gottlieb TM, Leal JFM, Seger R, Taya Y, Oren M (2002). Cross-talk between Akt, p53 and Mdm2: possible implications for the regulation of apoptosis. Oncogene.

[51] Martelli AM, Tazzari PL, Tabellini G, Bortul R, Billi AM, Manzoli L, Ruggeri A, Conte R, Cocco L (2003). A new selective AKT pharmacological inhibitor reduces resistance to chemotherapeutic drugs, TRAIL, all-trans-retinoic acid, and ionizing radiation of human leukemia cells. Leukemia.

[52] Kang J, Kisenge RR, Toyoda H, Tanaka S, Bu J, Azuma E, Komada Y (2003). Chemical sensitization and regulation of TRAIL-induced apoptosis in a panel of B-lymphocytic leukaemia cell lines. Brit J Haematol.

[53] Unni E, Koul D, Yung WKA, Sinha R (2005). Se-methylselenocysteine inhibits phosphatidylinositol 3-kinase activity of mouse mammary epithelial tumor cells in vitro. Breast Cancer Res.

[54] Li S, Zhou Y, Wang R, Zhang H, Dong Y, Ip C (2007). Selenium sensitizes MCF-7 breast cancer cells to doxorubicin-induced apoptosis through modulation of phospho-Akt and its downstream substrates. Mol Cancer Ther.

[55] Apel K, Hirt H (2004). Reactive oxygen species: Metabolism, oxidative stress, and signal transduction. Annu Rev Plant Bio.

[56] Chen T, Zheng W, Wong YS, Yang F (2008). Mitochondria-mediated apoptosis in human breast carcinoma MCF-7 cells induced by a novel selenadiazole derivative. Biomed Pharmacother.

[57] Lloyd DR, Phillips DH, Carmichael PL (1997). Generation of putative intrastrand cross-links and strand breaks in DNA by transition metal ion-mediated oxygen radical attack. Chem Res Toxicol.

[58] Liu S, Cao W, Yu L, Zheng W-J, Li L, Fan C, Chen T (2013). Zinc (II) Complexes Containing Bis-benzimidazole Derivatives as a New Class of Apoptosis Inducers That Trigger DNA damage-mediated P53 Phosphorylation in Cancer Cells. Dalton T.

[59] Tallarida RJ (2001). Drug synergism: Its detection and applications. J Pharmacol Exp Ther.

