# Supplementary material for: Selenocysteine derivative overcomes TRAIL resistance in melanoma cells: evidence for ROS-dependent synergism and signaling crosstalk
Source: Oncotarget. 2014 May 26;5(17):7431–45. doi: 10.18632/oncotarget.2008 (PMC4202134; doi:10.18632/oncotarget.2008)
Supplement: Supplementary file 1 [file oncotarget-05-7431-s001.pdf]

Selenocysteine derivative overcomes TRAIL resistance in melanoma cells: evidence for ROS-dependent synergism and signaling crosstalk

Supplementary Material

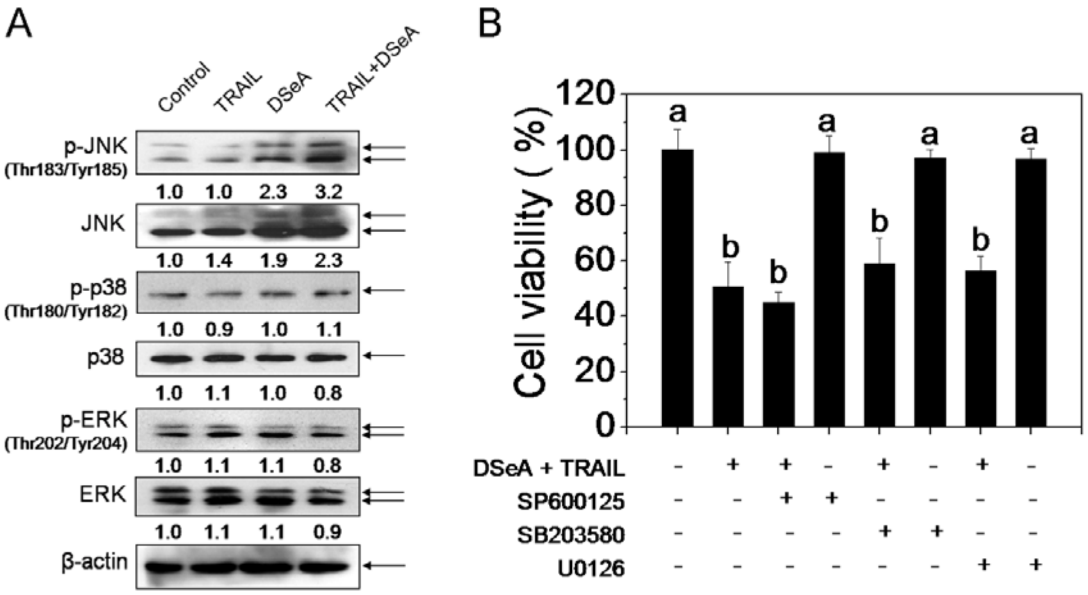

Figure S1: Contribution of MAPKs on co-treatment-induced growth inhibition in A375 cells. (A) Western bolt analysis of the effects of DSeA and/or TRAIL on the phosphorylation status and the expression level of MAPKs. Cells were preatreated with 20 µM DSeA for 24 h and then co-treated with 40 ng/ml TRAIL for another 24 h. (B) Effects of SP600125 (JNK inhibitor), SB203580 (p38 MAPK inhibitor) and U0126 (ERE inhibitor) on co-treatment-induced A375 cells growth inhibition. Cells were pretreated with 10 µM SP600125, SB203580 or U0126 1 h prior to the co-treatment. Cell viability was determined by MTT assay. All data are expressed as means ± SD of triplicates. Bars with different characters are statistically different at $p < 0.05$ level.

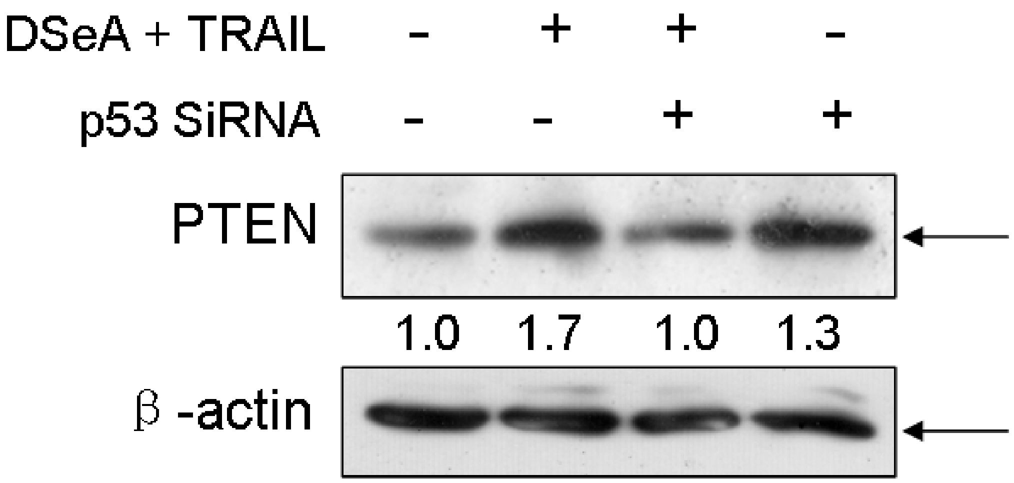

Figure S2: Protective effects of p53 siRNA on co-treatment-induced PTEN overexpression in A375 cells. Cells were pretreated with 50 nmol p53 siRNA for 24 h, and then treated in combination with DSeA and TRAIL.

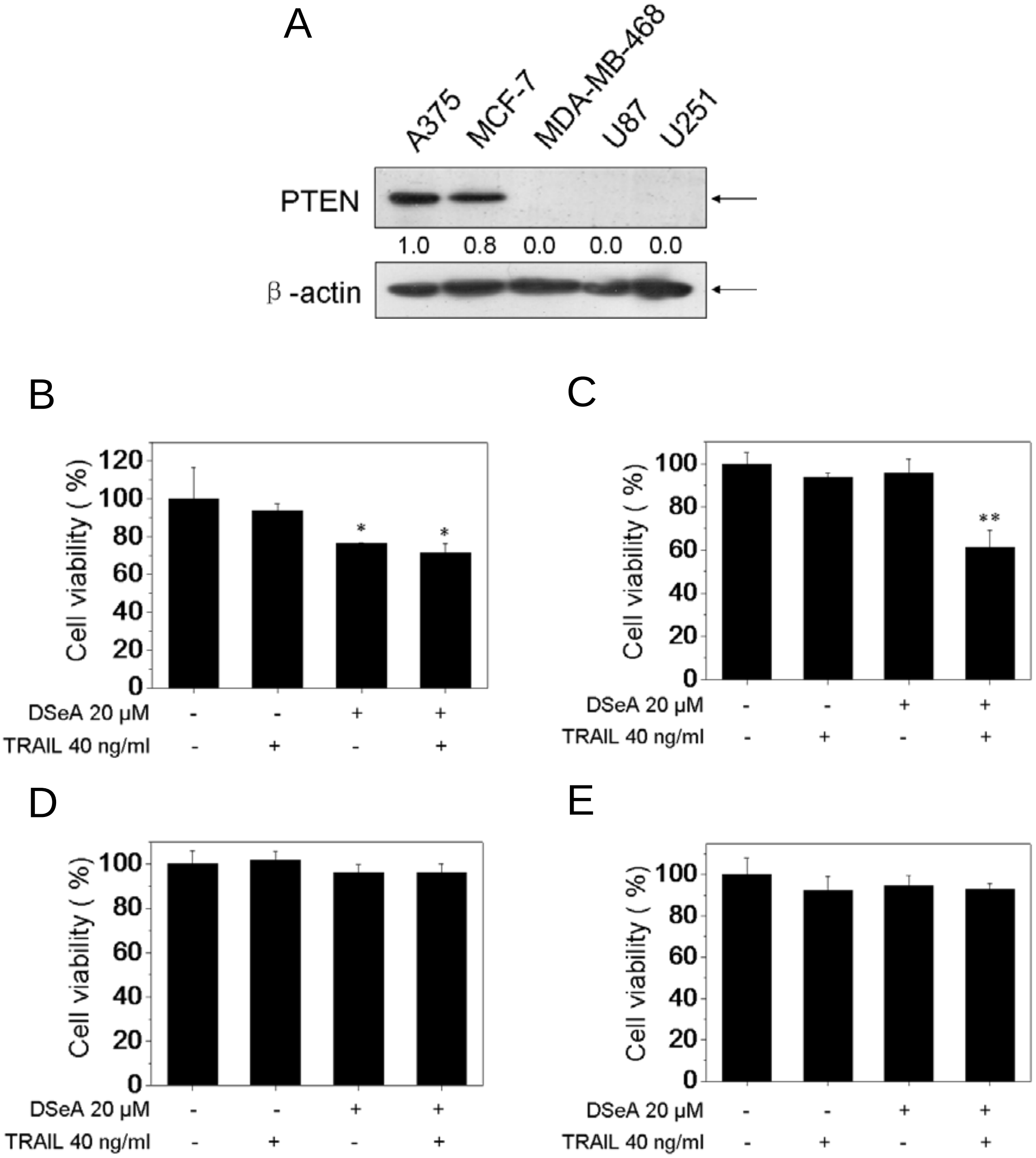

Figure S3: (A) The expression level of PTEN in the indicated cancer cell lines. The synergistic effects between DSeA and TRAIL in a PTEN-wild type MCF-7 cell line (B) and a panel of three PTEN-mutant human cancer cell lines, including MDA-MB-468 (C), U87 (D), U251 (E). Cells were pretreated with 20 μM DSeA for 24 h and then expose to 40 ng/ml TRAIL for another 24 h.

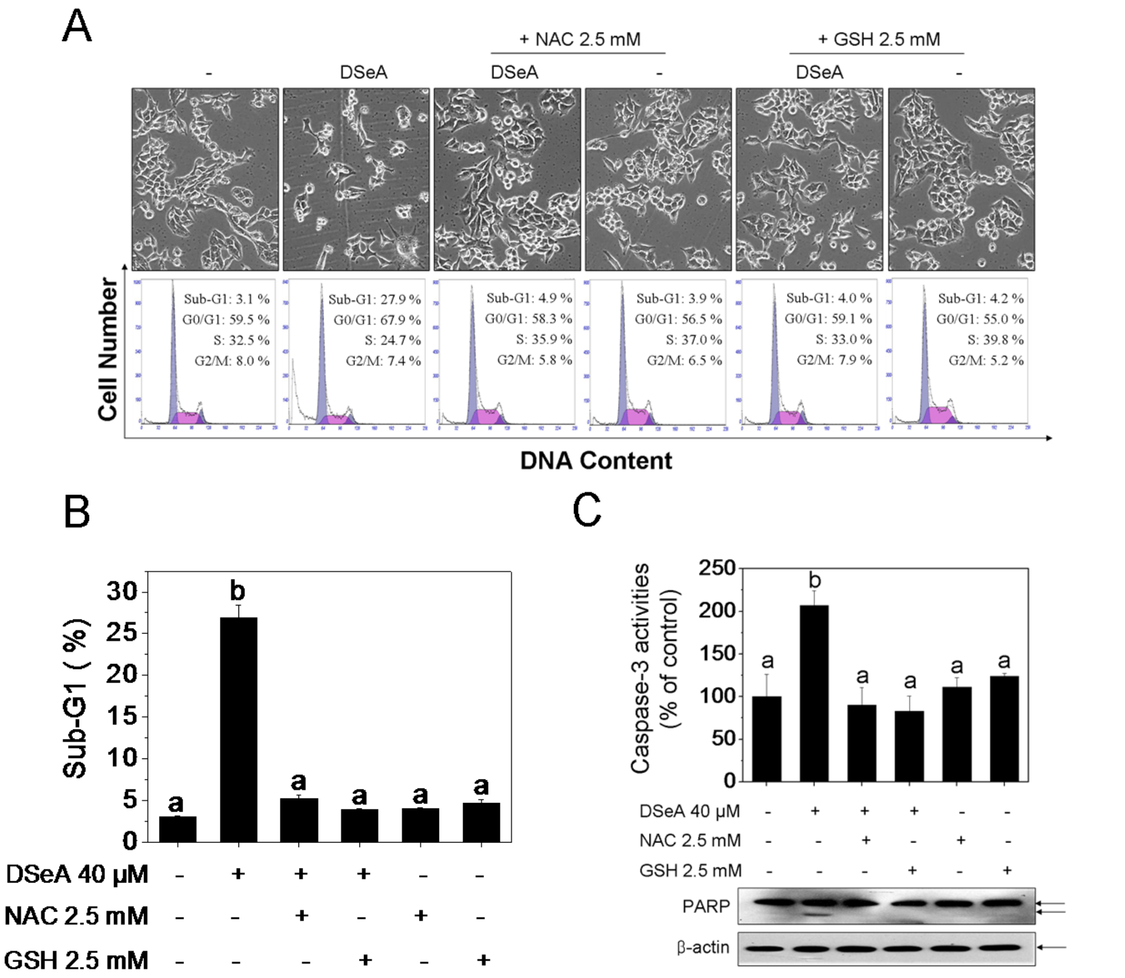

Figure S4: Roles of ROS in apoptotic cell death induced by DSeA in A375 cells. Inhibitory effects of NAC and GSH on DSeA-induced accumulation of sub-G1 cell population (A, B), activation of caspase-3 and PARP cleavage (C) in A375 cells. Cells were pretreated with 2.5 mM NAC or GSH for 1 h, and then co-treated with or without 40 μM DSeA for 24 h. Quantitative analysis of cell cycle distribution and apoptotic cell death were measured by flow cytometric analysis. Caspase activities were measured using synthetic fluorescent substrates for caspase-3. Bars with

different characters are statistically different at $p < 0.05$ level.

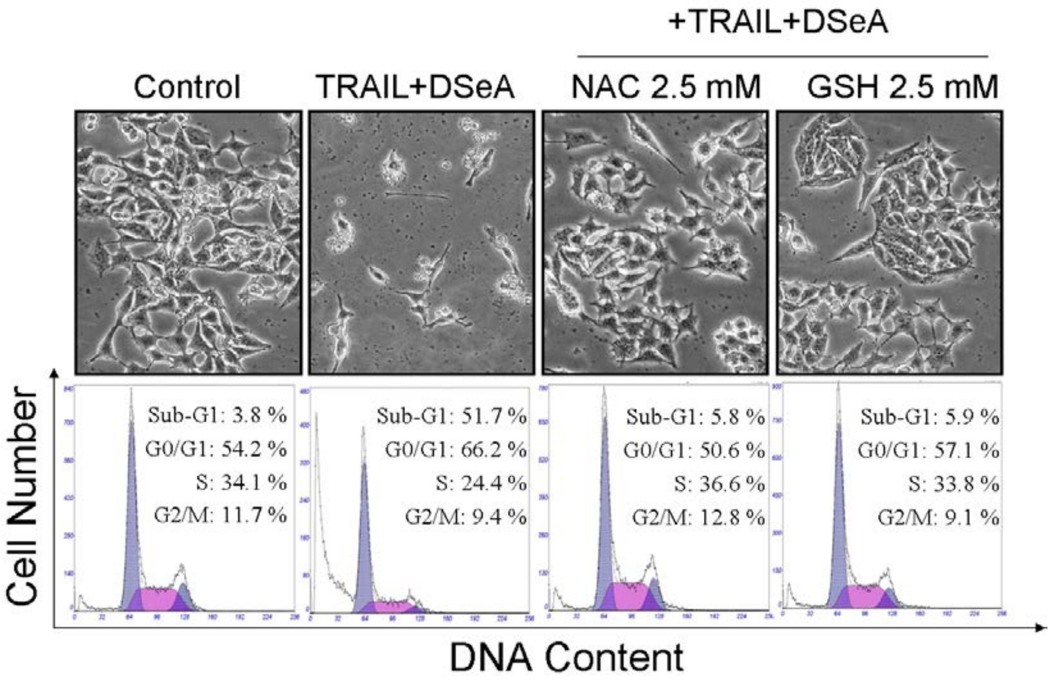

Figure S5: Inhibitory effects of NAC and GSH on co-treatment-induced accumulation of sub-G1 cell population.

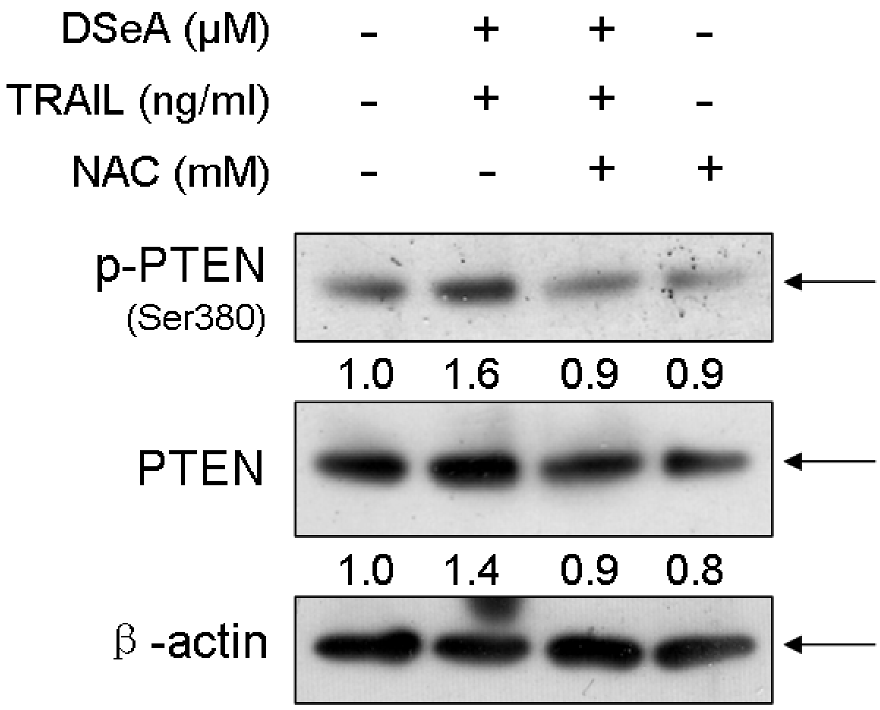

Figure S6: Protective effects of NAC on co-treatment-induced PTEN inactivation and overexpression. Cells were pretreated with or without 2.5 mM NAC for 1 h, and then co-treated with 20 μM DSeA for 24h, followed by treated with 40 ng/ml TRAIL for another 24 h.
